# Impact of the COVID-19 pandemic on the standard of care for patients with lysosomal storage diseases: A survey of healthcare professionals in the Fabry, Gaucher, and Hunter Outcome Survey registries

**DOI:** 10.1016/j.ymgmr.2021.100788

**Published:** 2021-08-04

**Authors:** Deborah Elstein, Roberto Giugliani, Joseph Muenzer, Jörn Schenk, Ida V.D. Schwartz, Christina Anagnostopoulou

**Affiliations:** a Independent consultant, Jerusalem, Israel; b Medical Genetics Service and Reference Center for Rare Diseases, Hospital de Clínicas de Porto Alegre, and Department of Genetics, UFRGS, Porto Alegre, Brazil; c University of North Carolina at Chapel Hill, Chapel Hill, NC, USA; d Takeda Pharmaceuticals Inc., Zurich, Switzerland

**Keywords:** Lysosomal storage disease, Fabry Outcome Survey, Gaucher Outcome Survey, Hunter Outcome Survey, COVID-19, Standard of care, COVID-19, coronavirus disease 2019, ERT, enzyme replacement therapy, FOS, Fabry Outcome Survey, GOS, Gaucher Outcome Survey, HCP, healthcare professional, HOS, Hunter Outcome Survey, IT, information technology, LSD, lysosomal storage disease, MPS II, mucopolysaccharidosis II

## Abstract

The impact of the COVID-19 pandemic on the standards of care of patients with lysosomal storage diseases and the needs of their healthcare providers were explored using a 12-question survey. Overall, 80/91 respondents (88%) indicated that the pandemic had negatively affected standards of care. With increased reliance on telemedicine, the respondents highlighted the need for a personalized approach to care, direct and frequent communication with patients, and greater involvement of patients and caregivers.

## Highlights

• The COVID-19 pandemic has changed how physicians and patients interact.
• A survey explored the impact of these changes on the care of patients with lysosomal storage diseases.
• 88% of respondents noted that the pandemic had affected standards of care.
• Increased need for personalized care and direct communication was noted.
• Increased reliance on telemedicine was also highlighted.

## 1 Introduction

Lysosomal storage diseases (LSDs), including Fabry disease, Gaucher disease and mucopolysaccharidosis II (MPS II; Hunter syndrome), are rare metabolic diseases characterized by the accumulation of toxic materials within lysosomes due to a deficiency in the activity of the enzymes responsible for their degradation [1]. The accumulation of these substrates leads to progressive multisystemic disease and, in some cases, premature death. Current disease-specific standard of care for the treatment of LSDs includes intravenous enzyme replacement therapy (ERT), substrate reduction therapy, and pharmacological chaperones [2]. In the context of the coronavirus disease 2019 (COVID-19) pandemic, published patient surveys suggest that COVID-19 has had a negative impact on mental health and access to continued care and treatment among patients with LSDs [[3], [4], [5], [6], [7], [8], [9], [10]]. The associated changes in clinical practice and in the needs of healthcare professionals (HCPs) in this evolving environment, however, have not been explored.

This survey aimed to explore the impact of the COVID-19 pandemic on the standards of care of patients enrolled in the Fabry, Gaucher, and Hunter Outcome Surveys (FOS, GOS, and HOS, respectively), along with the needs of HCPs treating patients with LSDs. The role of the FOS, GOS, and HOS registries is to collect long-term, real-world evidence on the safety and efficacy of treatment and on the natural history of Fabry disease, Gaucher disease and MPS II. As such, these global registries offered an opportunity to survey the opinions of a large group of HCPs from across the world and to explore the changing environment of care for patients with complex and rare metabolic diseases, providing a snapshot of the impact of the pandemic on standards of care.

## 2 Materials and methods

A 12-question survey was developed using the online platform SurveyMonkey® and invitations to complete the survey were sent via e-mail to 252 active sites involved in the FOS (*n* = 91), GOS (*n* = 44), and HOS (*n* = 117) registries across 38 countries and five continents. Each site was asked to complete the survey only once. The survey was active for completion between 29 January and 7 March 2021. The responses received were analyzed and percentages were used to describe the frequency of responses to each question.

## 3 Results and discussion

In total, 92 survey responses were received (36.5% response rate) and most respondents were Principal Investigators (85.9%). The remainder of responses were received from Sub-Investigators, nurses or nurse specialists, study or research coordinators, and genetic counsellors. Overall, of those who responded, 54.3% were involved in HOS, 30.4% in FOS, and 15.2% in GOS. As might be expected for rare diseases, most sites (56.5%) had fewer than 10 active registry patients enrolled. Most sites also indicated that the COVID-19 pandemic had had a negative impact on patient enrollment (‘slight impact’, 29.3%; ‘moderate impact’, 19.6%; and ‘great impact’, 18.5%). This was expected because registry activities were limited at most sites at the height of the COVID-19 pandemic in 2020, and the impact on enrollment is compounded by the challenges of obtaining patient consent in the absence of face-to-face visits. It is also important to highlight that disruptions due to the pandemic, for example in data collection and entry, may have long-term impacts on registry data collection and LSD research.

**Fig. 1:**
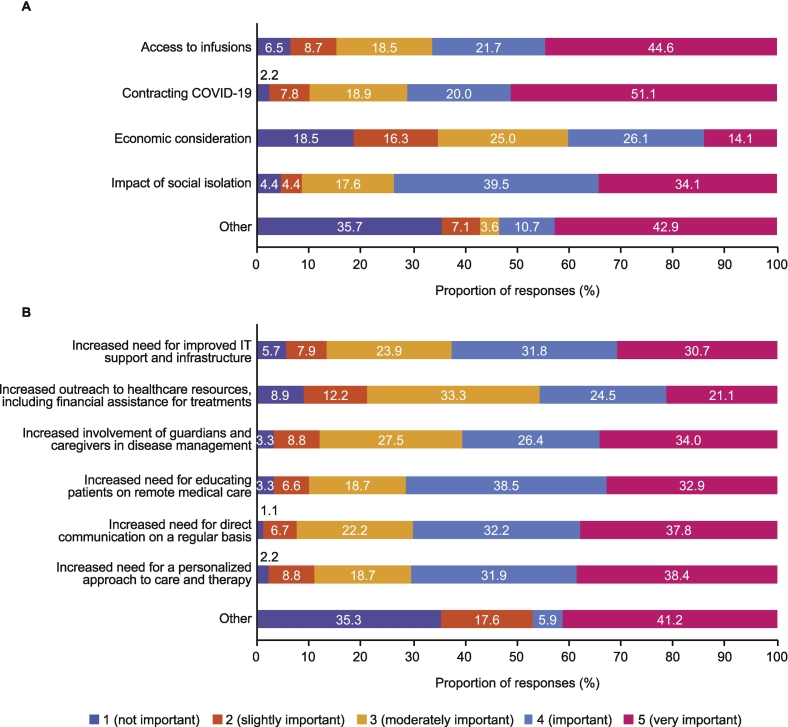
(**A**) Concerns of HCPs for their patients with LSDs regarding COVID-19 and (**B**) the needs of HCPs to enable the continued delivery of quality medical care to patients with LSDs in the context of extensive reliance on telemedicine, remote communication, and remote technology because of the COVID-19 pandemic. COVID-19 = coronavirus disease 2019; HCP = healthcare professional; IT = information technology; LSD = lysosomal storage disease.

Using a 5-point scale (1, not important and 5, very important), HCPs were asked to rank their concerns for their patients with LSDs. Most respondents considered contracting COVID-19, the impact of social isolation, and access to infusion or medications as important or very important (i.e. score of 4 or 5) (Fig. 1A). Other factors noted by respondents as free text entries included psychiatric effects, stress and fear, access to vaccination, continued treatment, optimum care, disease surveillance and disease management, and access to school, social, and community services. The concerns noted by HCPs for their patients in this survey appear to align with the results of published patient surveys [[3], [4], [5], [6], [7], [8], [9]]. Furthermore, most respondents were concerned that the risks associated with contracting COVID-19 for patients with LSDs may be higher than for the general population: 47.8% of respondents were ‘somewhat concerned’ and 34.8% of respondents were ‘greatly concerned’; however, 17.4% of respondents were ‘not concerned’ that risks may be higher in this patient population.

As might be expected and in line with recent reports [3,[5], [6], [7], [8], [9],^11^], HCPs noted that there had been some interruptions to treatment because of the COVID-19 pandemic: 38.0%, 32.6%, and 15.2% of respondents noted that < 1%, 1–10%, or > 10–20% of patients, respectively, had missed infusions. A small proportion of HCPs reported that patients had missed > 20–50% or > 50% of infusions (4.3% and 5.4% of respondents, respectively). In addition, when asked what percentage of missed infusions was acceptable, most HCPs (*n* = 62; 68.1%) deemed missing < 10% of infusions as acceptable, with a minority of respondents selecting missing 10–30% (*n* = 12; 13.2%), > 30–50% (*n* = 2; 2.2%), or > 50% (*n* = 4; 4.4%) of infusions as acceptable. Recent publications suggest that access to infusions during the COVID-19 pandemic may have been disrupted for several reasons, including, for example, issues at the hospital or infusion site, self-isolation or caregiver isolation due to COVID-19 symptoms, or personal decision due to fear of infection [[4], [5], [6], [7]]. Although the long-term impact of treatment interruptions among patients with LSDs due to the COVID-19 pandemic has yet to be examined, previous studies have suggested that treatment interruptions can be associated with aggravation of symptoms and worsening of clinical outcomes; it has also been reported that recommencement of therapy may not fully reverse the observed clinical decline [[12], [13], [14], [15], [16]].

When asked whether patients had switched to oral therapies (when available) because of the challenges posed by the COVID-19 pandemic for intravenous ERT, only 4.4% of sites indicated that some patients had switched to oral therapy; 56.5% and 39.1% of respondents selected ‘no switches’ or ‘not applicable’, respectively. It is important to note that the greatest number of responses came from HOS sites, so the low proportion of HCPs reporting switches may reflect the fact that no disease-specific oral therapy is available for MPS II. The impact of missed infusions and the need to switch to oral therapies may also be low because of the availability of home infusions in some regions. Several publications have reported minimal disruption to treatment for patients receiving home infusions during the COVID-19 pandemic and have suggested that home therapy should be considered, if possible, to minimize interruptions and to ensure continued treatment during disruptive periods such as those caused by a global pandemic [3,5,[7], [8], [9],^13^,^17^].

Owing to the COVID-19 pandemic, there has been a major shift in how physicians interact with and treat their patients [18,19]. To explore the impact of the switch to telemedicine, using the same 5-point scale, HCPs were asked to consider what was important to allow the continued delivery of quality care given the reliance on remote patient communication and visits (Fig. 1B). This revealed that most HCPs considered the need for a personalized approach to care, direct and regular communication with patients, the increased involvement of guardians or caregivers, and the need for patient education as important or very important (i.e. score of 4 or 5) to allow the continued delivery of quality medical care. The need for improved information technology support and infrastructure was also noted. Although the pandemic has posed challenges for the delivery of quality medical care, some HCPs also noted as free text entries that the impact of the COVID-19 pandemic on the standards of care of patients had been minimal, owing to the availability of home care and robust telemedicine services in their region. However, 88.0% of respondents (80/91) overall indicated that the COVID-19 pandemic had negatively affected the standards of care in relation to LSDs (‘moderate impact’, 50.5%; ‘slight impact’, 31.9%; and ‘great impact’, 5.5%); 81.5% of respondents were also concerned that the reliance on virtual consultations had, to some extent, resulted in delays in diagnosis of LSDs. The changes in medical practice due to the COVID-19 pandemic, however, highlight the importance of robust telemedicine and provide an opportunity to leverage available healthcare technologies to optimize the delivery of personalized medicine for the future and to improve patient quality of life. This may include, for example, the greater involvement of patients and caregivers in disease monitoring via the use of digital applications and patient-reported outcome measures.

Limitations of this study included the variation in the number of responses from HOS, FOS and GOS sites (54%, 30%, and 15% of respondents were from HOS, FOS and GOS sites, respectively). This may have had an impact on responses to multiple questions: for example, economic considerations and the role of guardians and caregivers in disease management are likely to vary depending on the disease. This variation arose from a difference in the number of active sites in each registry, but also a slight difference in response rates (43%, 31%, and 32% for HOS, FOS, and GOS, respectively). Reasons for the higher response rate of HOS active sites compared with FOS and GOS sites are not clear, but it is possible that reassuring reports in the literature regarding the risks and impact of COVID-19 in patients with Fabry disease and Gaucher disease, in addition to overlap of Investigators across registries, may have contributed to slightly lower response rates from the FOS and GOS sites [6,10,17,[20], [21], [22]]. Furthermore, information on the number of COVID-19 infections observed at the sites was not captured in this survey, and respondents were not asked to provide their location, so regional differences in experiences of the pandemic could not be accounted for.

## 4 Conclusions

This global survey canvassed the opinions of HCPs involved in the care of patients with LSDs during the COVID-19 pandemic and revealed that the pandemic has led to some disruptions affecting access to care and treatment for patients. The results highlighted the concerns of HCPs for their patients (including risk of infection, access to treatment, and social isolation), the need for personalized medical care, regular and direct communication with patients, and the increased involvement of guardians and caregivers in disease management for the continued delivery of medical care. Furthermore, most HCPs reported that the pandemic had negatively affected standards of care for patients with LSDs. Looking forward, it is important to consider the lessons learnt and how these can be applied to future standards of care for patients with LSDs and other rare metabolic diseases.

## Author contributions

Deborah Elstein: Conceptualization, Formal Analysis, Writing – Original draft.

Roberto Giugliani: Conceptualization, Formal Analysis, Writing – Original draft.

Joseph Muenzer: Conceptualization, Formal Analysis, Writing – Original draft.

Ida V.D. Schwartz: Conceptualization, Formal Analysis, Writing – Original draft.

Jörn Schenk: Conceptualization, Formal Analysis, Writing – Original draft.

Christina Anagnostopoulou: Conceptualization, Formal Analysis, Writing – Original draft.

## Funding

The FOS, GOS, and HOS registries are sponsored and funded by 10.13039/100007343Shire (a Takeda company). Survey data collection and analysis was performed by Oxford PharmaGenesis under the direction of the authors, and was funded by Takeda Development Center Americas, Inc. Medical writing support was funded by Takeda Development Center Americas, Inc. The final decision to submit the manuscript for publication was made by the authors.

## Declaration of Competing Interest

**D.E.** has no conflicts of interest to disclose. D.E. is a member of the GOS Steering Committee.

**R.G.** has received consulting fees, fees for service, speaker fees and/or travel expenses from and/or participated in advisory boards for Abeona Therapeutics, Alnylam Pharmaceuticals, Amicus Therapeutics, BioMarin Pharmaceutical, Chiesi Farmaceutici, Denali Therapeutics, Inventiva Pharma, Janssen Pharmaceuticals, JCR Pharmaceuticals, Novartis, Orphan Disease Center, REGENXBIO, Sanofi Genzyme, Sigilon, Swedish Orphan Biovitrum, Takeda, and Ultragenyx Pharmaceutical. He has performed contracted research for Allievex, Amicus Therapeutics, Idorsia Pharmaceuticals, JCR Pharmaceuticals, Lysogene, Sanofi Genzyme, Takeda, and Ultragenyx Pharmaceutical. R.G. is also the chair of the FOS Steering Committee.

**J.M.** has received consulting fees from and/or participated in advisory boards for bluebird bio, Denali Therapeutics, Homology Medicines, JCR Pharmaceuticals, REGENXBIO, Sanofi Genzyme, and Takeda. He is a Principal Investigator for phase 1/2 and phase 2/3 intrathecal enzyme replacement therapy trials for the neuronopathic form of MPS II (sponsored by Shire, a Takeda company), a phase 1/2 gene editing trial for adults with MPS II (sponsored by Sangamo Therapeutics), a phase 1/2 intravenous enzyme replacement therapy trial for MPS IIIA (sponsored by Swedish Orphan Biovitrum), and a phase 1/2 intravenous enzyme replacement therapy trial for MPS II (sponsored by Denali Therapeutics). J.M. is also the chair of the HOS Steering Committee.

**I.V.D.S.** has received consulting fees, fees for service, speaker fees and/or travel expenses from and/or participated in advisory boards for Alnylam Pharmaceuticals, BioMarin Pharmaceutical, Janssen Pharmaceuticals, Pfizer/Protalix Biotherapeutics, Sanofi Genzyme, Takeda, and Ultragenyx Pharmaceutical. She has performed contracted research for Sanofi Genzyme, Takeda, and Ultragenyx Pharmaceutical. I.V.D.S. is also the chair of the GOS Steering Committee.

**J.S.** is a full-time employee of Takeda Pharmaceuticals Inc. and is a stockholder of Takeda Pharmaceuticals Company Limited.

**C.A.** is a full-time employee of Takeda Pharmaceuticals Inc. and is a stockholder of Takeda Pharmaceuticals Company Limited.
